# A cross-sectional survey of soil-transmitted helminthiases in two Myanmar villages receiving mass drug administration: epidemiology of infection with a focus on adults

**DOI:** 10.1186/s13071-017-2306-2

**Published:** 2017-08-04

**Authors:** Julia C. Dunn, Alison A. Bettis, Nay Yee Wyine, Aye Moe Moe Lwin, Soe Thiha Lwin, Khine Khine Su, Myint Myint Sein, Aung Tun, Nay Soe Maung, Roy M. Anderson

**Affiliations:** 1 Department of Infectious Disease Epidemiology, School of Public Health, Faculty of Medicine, Imperial College London, W2 1PG, London, UK; 2 London Centre for Neglected Tropical Disease Research, London, UK; 3 University of Public Health, Myorma Kyaung Street, Yangon, 11131 Myanmar; 4 Defence Services Medical Academy, Pyay Road, Mingaladon, Yangon, 11021 Myanmar; 5 Ministry of Health and Sports, Nyapyitaw, Myanmar

**Keywords:** Soil-transmitted helminths, Neglected tropical diseases, Myanmar, Cross-sectional survey, Mass drug administration

## Abstract

**Background:**

Soil-transmitted helminths (STH) are still highly prevalent in southeast Asia. The country of Myanmar has had ongoing mass drug administration (MDA) programmes since 2003 in an attempt to control STH and reduce STH-related morbidities. Whilst the MDA programmes have reported high nationwide coverage, there have been no epidemiological surveys that included measurements from adults. This paper details three cross-sectional surveys that took place over the course of a year in two villages endemic for STH and receiving MDA in lower Myanmar.

**Results:**

At baseline, 27.81% of participants were infected with at least one type of STH. The most prevalent STH was *Trichuris trichiura* (18.12%) followed by hookworm (8.71%) and *Ascaris lumbricoides* (5.34%). Most infections were of low intensity, measured by eggs per gram of faeces (EPG). Gender stratification revealed that *A. lumbricoides* prevalence was significantly higher in females, whereas hookworm prevalence was significantly higher in males. The distribution of EPG in the study sample was highly overdispersed, suggesting that most people release few eggs whereas a few people release many eggs. Adults harbour a major proportion of the overall STH burden; 65.15% of STH infections were harboured by adults.

**Conclusions:**

STH infection remains at medium prevalence in the study villages despite past and recent MDA. Recorded prevalence of STH in school-aged children has not substantially decreased since the last monitoring and evaluation activities in Myanmar in 2013. Analyses suggest that adults are a major contributor to the total STH prevalence and EPG burden, probably perpetuating transmission.

**Electronic supplementary material:**

The online version of this article (doi:10.1186/s13071-017-2306-2) contains supplementary material, which is available to authorized users.

## Background

Soil-transmitted helminths (STH) are a group of intestinal nematodes with direct life-cycles. There are four main species that cause disease in humans; *Ascaris lumbricoides* (roundworm), *Trichuris trichiura* (whipworm) and the hookworms (*Necator americanus* and *Ancylostoma duodenale*). The World Health Organization (WHO) designates STH infections as neglected tropical diseases (NTDs) and estimates that they affect more than 1.4 billion people worldwide [1]. STH infection causes low mortality, but chronic and repeated infection from childhood can lead to malnutrition, mental deficits, and physical and intellectual growth impairment [2]. An estimated 5.18 million disability-adjusted life years (DALYs) have been attributed to STH infection [3].

The control method for STH recommended by the WHO is mass drug administration (MDA) to those most likely to suffer heavy infection and the concomitant morbidity. The current strategy consists of treating school-aged children (SAC, aged 5–14 years old) and preschool-aged children (pre-SAC, aged 2–4 years old), regardless of their infection status, with the anthelminthic drugs albendazole or mebendazole [4]. The London Declaration on NTDs included a pledge that these two anthelminthic drugs will be donated by the manufacturing companies (GlaxoSmithKline and Johnson & Johnson, respectively) until at least 2020 [5]. The WHO target for STH MDA is for endemic countries to achieve 75% treatment coverage of pre-SAC and SAC by 2020 [6]. Global coverage has greatly increased over the past decade, but has not yet reached the 75% goal. Recent mathematical modelling studies of STH transmission and the effect of MDA on transmission have concluded that STH cannot be eliminated by MDA programmes targeting pre-SAC and SAC alone, adults must also be treated and at high coverage levels, especially in areas where hookworm is the dominant infection [7, 8].

A spatial epidemiology review by Pullan et al. [3] concluded that the region with the highest prevalence of STH infection is southeast Asia. Myanmar is a low to middle income country in the southeast Asia region and has a history of epidemiological research on STH infection and control [9]. Myanmar currently has government-run MDA programmes in place for both STH and lymphatic filariasis (LF) treatment. STH MDA takes place in August, treating all SAC with albendazole. LF MDA takes place in December or January (dependent on when the drugs arrive in-country), treating the whole eligible community with albendazole and diethylcarbamazine citrate (DEC). From these overlapping programmes, pre-SAC and adults receive annual albendazole treatment and SAC receive biannual albendazole treatment. The most recent data reported to the WHO PCT databank by the Myanmar government have national pre-SAC MDA coverage at 95.45% and SAC coverage at 99.18% for 2015 [10]. In 1984, Hlaing et al. [11] found *A. lumbricoides* in 77.1% of a village community in the Yangon (then Rangoon) region. Since the Hlaing et al. study, all epidemiology studies in Myanmar have focussed on SAC. A national survey conducted in 2002–2003 recorded 48.5% *A. lumbricoides*, 57.5% *T. trichiura* and 6.5% hookworm in 1000 SAC [12]. MDA began immediately after this survey in 2003 [10]. By 2013, these had decreased to 5.8% *A. lumbricoides*, 18.6% *T. trichiura* and 0.3% hookworm [13]. Another study, also in 2013, focussed on SAC in the Yangon Region, found similar prevalence of each STH [14].

The epidemiology study presented in this paper aims to examine the current epidemiological pattern of STH infection and the effect of MDA in two villages in lower Myanmar. This paper details the methods employed in the study but focusses on the results of the first cross-sectional study survey only. This includes the demography of the study villages, determining the prevalence and mean infection intensity (measured by eggs per gram of faeces, EPG) of each STH, the age distribution of STH infection and how infection is dispersed within the study sample. Particular attention is given to infection in adults, as this age group has been under sampled for STH research in Myanmar since MDA started. The primary aims of this epidemiological study are to provide a more complete population-based picture of current STH infection after a period of exposure to MDA and to further understand the factors that result in transmission persistence despite high reported MDA coverage. The STROBE framework for observational studies was followed for this manuscript and the statement is included as Additional file 1.

## Methods

### Study sites

**Fig. 1:**
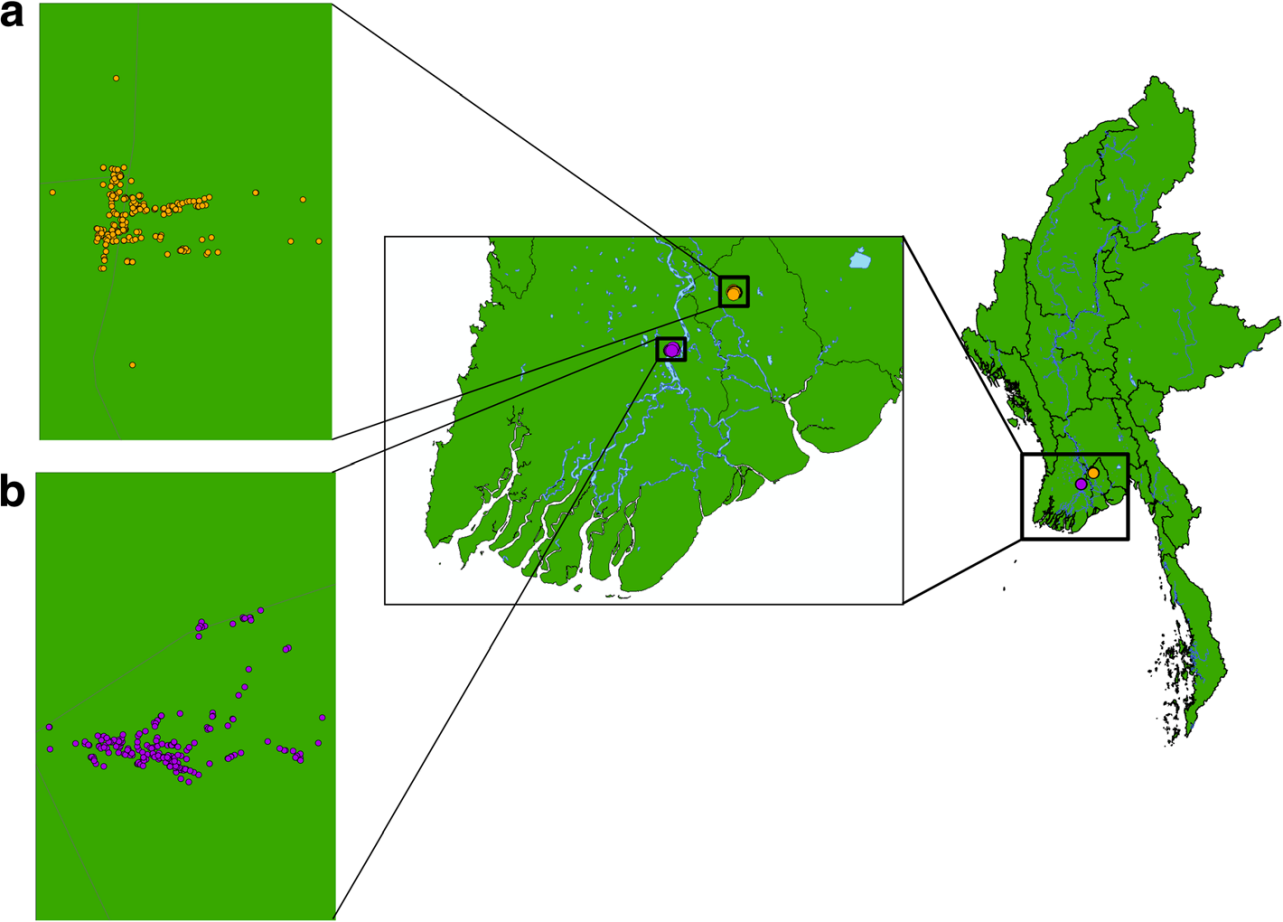
Map of Myanmar and the study villages. **a** Udo village, Taikkyi township, Yangon Region. **b** Kyee Kan Theik village, Nyaung Don township, Ayeyarwaddy Region. Circles indicate individual households

Study sites were chosen by the criteria of (i) their accessibility throughout the year; (ii) a minimum population size of 600 people; (iii) whether the population was stable in composition and size; and (iv) the level of cooperation provided by village leaders and health personnel. Udo village, Taikkyi township, Yangon Region and Kyee Kan Theik village, Nyaung Don township, Ayeyarwaddy Region were selected as study sites. Udo village is in a peri-urban area, located on the Pyay Road that runs north from Yangon to Pyay. Kyee Kan Theik village is in a rural area and is at high risk of flooding during the rainy season due to its proximity to the Irrawaddy River. Both villages are classed as part of the “delta region” [15] where the climate is tropical and humid. The mean annual temperature of the delta region is 32 °C and the average annual rainfall is 2500 mm; the rainy season lasts from May to early October. There are approximately 237 households in Udo village, with an average of 4.9 people per household, and 355 households in Kyee Kan Theik village, with an average of 4.5 people per household. The main form of employment for both villages is farming and agriculture (as is typical in much of rural Myanmar), followed by self-employment in small shops and family-run businesses. Figure 1 shows the location of the villages in Myanmar and the location of the village households as determined by Global Positioning System (GPS) coordinates.

### Study participants

In June 2015, a demographic survey and census were completed in the two study villages. Village residents had the study methods and aims described to them, and were asked if they would consent to participate in the study. Individuals that fulfilled the inclusion criteria (informed written consent provided, over two years of age, not pregnant/breastfeeding and permanent residents of the village) were grouped into households, and households were chosen for the study by random selection. Children under two years old and pregnant women were excluded as albendazole has not been approved for use in these groups [16].

Since there was no prior data on STH prevalence in these villages, a sample size was determined by the number of stool specimens the laboratory team could process during the allotted study time and, factoring in an estimated 20% loss to follow-up, this came to approximately 1000 people. Using the final achieved sample size and recorded STH prevalence, the error calculated was 3.29% at the two-sided 5% significance level (Additional file 2). Study participation (participants with completed surveys and a recorded Kato-Katz result) for the first, second and third survey was 72.88%, 68.17% and 67.45%, respectively. Whilst the study methods were clearly explained before consent was sought, some participants experienced fatigue with the study and did not wish to take albendazole or collect faeces, at which point they were classed as “lost to follow-up” (Additional file 3: Figure S1). All participants had a unique ID code assigned to them so that their data could be longitudinally linked between surveys and remain confidential.

### Data collection

The study comprised of three data collection surveys, in August 2015 (first survey), December 2015 (second survey) and June 2016 (third survey) (Additional file 4: Figure S2). The first two surveys coincided with the government MDA rounds and the final survey was conducted two months before the MDA round due to study time restrictions. Participants that were randomly selected after the census stage were followed up in each survey to collect longitudinal data. In each study survey, participants were asked to complete a questionnaire on STH knowledge, attitudes and practice (KAP) and MDA compliance (“epidemiology survey”). Each participant provided a small stool specimen which was assessed for STH infection by the Kato-Katz method [17]. All thick-smear slides were prepared and read on the same day as collection. The slides were read within one hour of preparation to identify hookworm eggs, and then read again for *A. lumbricoides* and *T. trichiura* eggs one to two hours after slide preparation to allow the slides to clear. Due to time constraints, only one slide was examined for each participant. For quality control purposes, a random 10% of slides were re-read by a second laboratory technician. Of the re-read slides, 97.87%, 80.85% and 87.23% were in agreement (within the same WHO intensity group) for *A. lumbricoides*, *T. trichiura* and hookworm, respectively. The species of hookworm was not differentiated. STH infection data were recorded as egg counts and were multiplied by 24 to give EPG. All participants were treated with anthelminthics after stool collection; albendazole only in the first survey and the third survey, albendazole and DEC in the second survey. The rest of the community were treated at the same time as the study surveys by the government MDA programme.

### Statistical analysis

Data for the following analyses were from all participants with a recorded Kato-Katz result in the first survey (August 2015). Data from the two villages were merged and analysed together. Subsequent reports will focus on the longitudinal data from all three surveys. ArcGIS software (ArcMap 10.2.2, ESRI, Redlands, CA, USA), Microsoft Excel 2010 (Microsoft, Redmond, WA, USA) and RStudio (R version 3.0.1, Vienna, Austria) were used to generate the figures. RStudio was also used for the following statistical analyses. Confidence intervals (CIs) for mean prevalence within age groups were calculated using the Clopper-Pearson method. Adjusted bootstrap percentiles (BCa) for mean EPG within age groups were calculated using the “*boot*” package. EPG results were grouped by the WHO recommended intensity groupings into low, medium and high intensity of infection [4]. Chi-square tests were used to analyse association between categorical variables (e.g. gender and age group) and binary (e.g. STH prevalence) or categorical variables. Kruskall-Wallis tests were used to analyse associations between categorical variables and negative binomially distributed continuous variables (EPG). The null hypothesis for both tests was that there would be no statistically significant difference in the outcome variable between the groups of the explanatory variable. The statistical significance level was set at *P* < 0.05. To analyse the relationship between egg count (the number of eggs counted in the faecal sample for each participant) mean and variance, egg count data were log-transformed. All means presented are arithmetic means, unless stated otherwise.

## Results

### Participant characteristics

Overall, there were 712 participants from 251 households who had Kato-Katz data in the first survey. The age distribution of the sampled participants closely matches the overall demography of the study villages (Additional file 5: Figure S3). However, in comparison to the national age distribution as reported by the 2014 Population and Housing Census of Myanmar [18], young adults, especially 15–19 year olds, were under sampled and 2–9 year olds were over sampled. Table 1 presents demographic and socioeconomic characteristics of sampled study participants.

**Table 1:** Study participant characteristics

Characteristic		Udo village		Kyee Kan Theik village	
		*n*	%	*n*	%
Individuals		305	100	407	100
Sex	Male	131	42.95	196	48.16
	Female	174	57.05	211	51.84
Age group (years)	2–4	20	6.56	33	8.11
	5–14	60	19.67	98	24.08
	15–24	36	11.80	41	10.07
	25–39	67	21.97	107	26.29
	40+	122	40.00	128	31.45
Households		114	100	137	100
Household monthly income^a^	< 50,000 MMK	7	6.14	36	26.28
	50,000–100,000 MMK	55	48.25	64	46.72
	100,000–500,000 MMK	48	42.11	31	22.63
	> 500,000 MMK	2	1.75	3	2.19
	Do not know/refused to answer	2	1.75	3	2.19
Overall household size^b^	1–4	53	46.49	79	57.66
	5–8	58	50.88	52	37.96
	9+	3	2.63	6	4.38
Main source of water	Bottled water	5	4.39	0	0
	Piped water (into home/compound)	3	2.63	8	5.84
	Well (protected/unprotected)	3	2.63	0	0
	Rain water (covered/uncovered)	1	0.88	0	0
	Surface water	0	0.00	99	72.26
	Tubewell/borehole	100	87.72	27	19.71
	Public tap	1	0.88	1	0.73
	Other	1	0.88	0	0
	Do not know/refused	0	0	2	1.46
Type of toilet	Bucket	2	1.75	11	8.03
	Composting toilet	13	11.40	10	7.30
	Flush toilet	56	49.12	59	43.06
	Open pit	1	0.88	2	1.46
	Pit latrine with slab	30	26.32	46	33.58
	VIP	12	10.53	9	6.57

^a^Cumulative income from all members of the household

^b^Including non-participants

*Abbreviations*: MMK, Myanmar kyats (1 USD to 1111 MMK, June 2015); VIP, ventilated improved pit latrine

### Prevalence of STH infection

The prevalence of infection with at least one STH was 27.81% (198/712). *Trichuris trichiura* was the most prevalent STH in both villages with a prevalence of 18.12% followed by hookworm (8.71%) and *A. lumbricoides* (5.34%). Prevalence of *A. lumbricoides* and *T. trichiura* peaked in the 5–14 years old age group and decreased over the older age groups (Table 2). However, prevalence of hookworm was lower in the pre-SAC and SAC age groups, increasing with age and peaking in 25–39 years old. The difference in prevalence between age groups was statistically significant for each STH species (*A. lumbricoides*: *χ* ^2^ = 11.45, *P* < 0.05; *T. trichiura*: *χ* ^2^ = 24.97, *P* < 0.0001; hookworm: *χ* ^2^ = 18.23, *P* < 0.01). Males and females had similar prevalence of any STH infection (28.44 and 27.27%, respectively, *χ* ^2^ = 0.07, *P* = 0.79). The difference in prevalence between males and females was statistically significant for *A. lumbricoides* (higher prevalence in females, *χ* ^2^ = 5.41, *P* < 0.05.) and hookworm (higher prevalence in males, *χ* ^2^ = 13.99, *P* < 0.001) but not for *T. trichiura* (*χ* ^2^ = 1.73, *P* = 0.19).

**Table 2:** Number of participants (*n*), prevalence (%) and infection intensity of each soil-transmitted helminth

			Any STH		*Ascaris lumbricoides*			*Trichuris trichiura*			Hookworm		
		*n*	*n*	% (95% CI)^a^	*n*	% (95% CI)	Mean EPG (95% CI)	*n*	% (95% CI)	Mean EPG (95% CI)	*n*	% (95% CI)	Mean EPG (95% CI)
Overall		712	198	27.81 (24.54–31.26)	38	5.34 (3.80–7.25)	1173.40 (610.36–2712.98)	129	18.12 (15.36–21.15)	66.27 (45.97–103.08)	62	8.71 (6.74–11.02)	33.88 (20.56–73.34)
Sex	Male	385	93	28.44 (23.61–33.66)	10	3.06 (1.48–5.55)	203.82 (72.36–592.41)	52	15.90 (12.11–20.32)	42.20 (26.47–69.77)	43	13.15 (9.68–17.30)	63.78 (35.89–145.84)
	Female	327	105	27.27 (22.88–32.01)	28	7.27 (4.89–10.34)	1996.92 (947.23–5134.87)	77	20.00 (16.12–24.35)	86.71 (52.28–154.21)	19	4.94 (3.00–7.60)	8.48 (4.80–14.96)
Age group (years)	2–4	53	12	22.64 (12.28–36.21)	3	5.66 (1.18–15.66)	2416.75 (127.70–11,117.89)	9	16.98 (8.07–29.80)	62.49 (27.62–123.62)	2	3.77 (0.46–12.98)	47.09 (0.00–141.28)
	5–14	158	57	36.08 (28.6–44.09)	16	10.13 (5.90–15.92)	3746.43 (1524.27–10,976.88)	48	30.38 (23.32–38.19)	156.46 (92.96–283.56)	2	1.27 (0.15–4.50)	2.13 (0.00–7.59)
	15–24	77	24	31.17 (21.09–42.74)	5	6.49 (2.14–14.51)	849.35 (141.82–3466.98)	16	20.78 (12.37–31.54)	141.82 (34.60–469.19)	9	11.69 (5.49–21.03)	21.82 (9.66–45.29)
	25–39	174	48	27.59 (21.09–34.86)	4	2.30 (0.63–5.78)	71.45 (15.72–272.93)	28	16.09 (10.97–22.41)	17.52 (10.90–27.81)	22	12.64 (8.10–18.51)	66.76 (23.59–257.37)
	40+	250	57	22.80 (17.75–28.51)	10	4.00 (1.93–7.23)	150.43 (39.46–434.28)	28	11.20 (7.57–15.78)	20.74 (11.33–41.75)	27	10.80 (7.24–15.32)	31.97 (16.90–62.94)

^a^% represents the percentage positive in each group

*Abbreviations*: CI, confidence interval; EPG, eggs per gram of faeces; STH, soil-transmitted helminth

### Intensity of STH infection

Intensity of infection of each STH was recorded as EPG of faeces. The distribution of intensity of infection over the age groups closely resembles the patterns of prevalence (Table 2). *Ascaris lumbricoides* and *T. trichiura* mean EPG peaked in the 5–14 age group at 3746.43 EPG and 156.46 EPG, respectively, and decreased in the older age groups. Hookworm intensity peaked in the 25–39 age group at 66.76 EPG but, as opposed to prevalence, EPG was also relatively high in the 2–4 age group. The difference in mean EPG was statistically significant for all age groups (*A. lumbricoides*: *χ* ^2^ = 12.21, *P* < 0.05; *T. trichiura*: *χ* ^2^ = 27.38, *P* < 0.0001; hookworm: *χ* ^2^ = 17.97, *P* < 0.01). Mean EPG was significantly higher in females for *A. lumbricoides* (*χ* ^2^ = 6.42, *P* < 0.05) and significantly higher in males for hookworm (*χ* ^2^ = 15.19, *P* < 0.0001). There was no significant difference in *T. trichiura* EPG between genders (*χ* ^2^ = 2.13, *P* = 0.14). Most *T. trichiura* and hookworm infections were classed in the low intensity group (91.47 and 98.39%, respectively). However, *A. lumbricoides* infections were mostly in the low (42.11%) and medium (47.37%) infection intensity groups.

### Parasite distribution

**Fig. 2:**
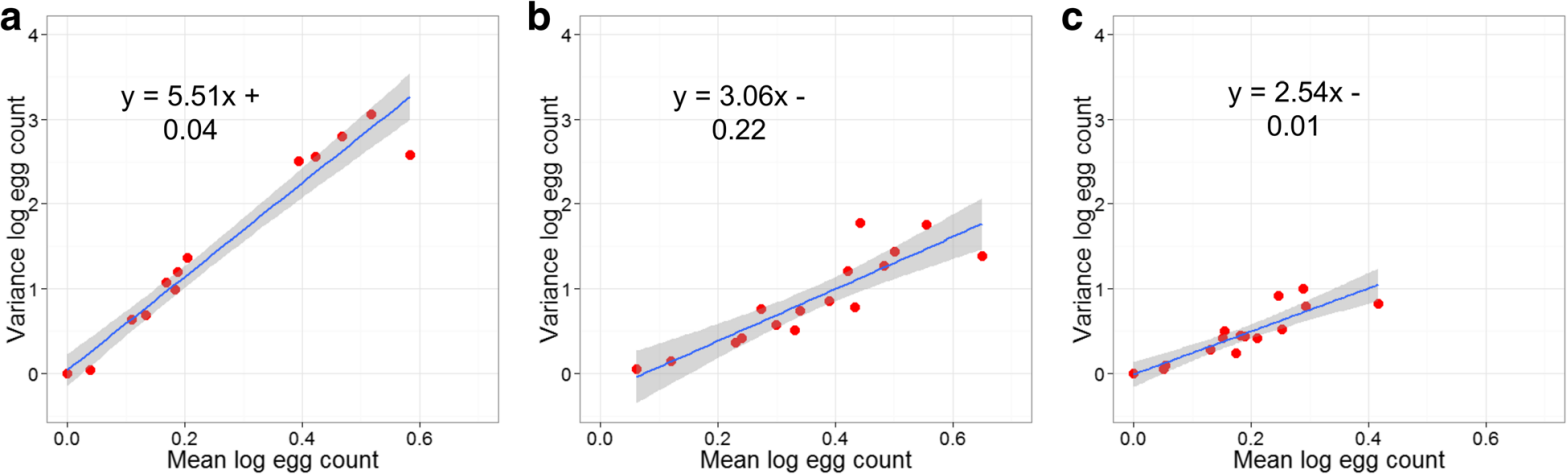
Mean log faecal egg counts against log faecal egg count variance. **a** *Ascaris lumbricoides*. **b** *Trichuris trichiura*. **c** Hookworm. Each data point represents a five-year age group of all participants with Kato-Katz data at baseline (*n* = 712). The shaded areas denote the standard error limits. Lines of best fit and formulae derived by linear regression

The distribution of STH eggs in the study sample was overdispersed; for each species, the variance in egg counts (the number of eggs counted in the faecal sample for each participant) within five-year age groups was consistently larger than the mean egg count (Fig. 2). When plotting the relationship between egg count mean and variance, if the linear regression line gradient coefficient is larger than one (or larger than zero when the data have been log-transformed) then the distribution is overdispersed, since for a Poisson (i.e. random) distribution the variance equals the mean value. An approximately linear relationship exists between the logarithm of the mean egg count and the logarithm of the variance of egg count. This is the pattern predicted by a negative binomial distribution for the EPG counts. In such plots, gradient values much greater than unity represent high degrees of aggregation in EPG within the sampled people within each age group.

### STH burden in adults

**Fig. 3:**
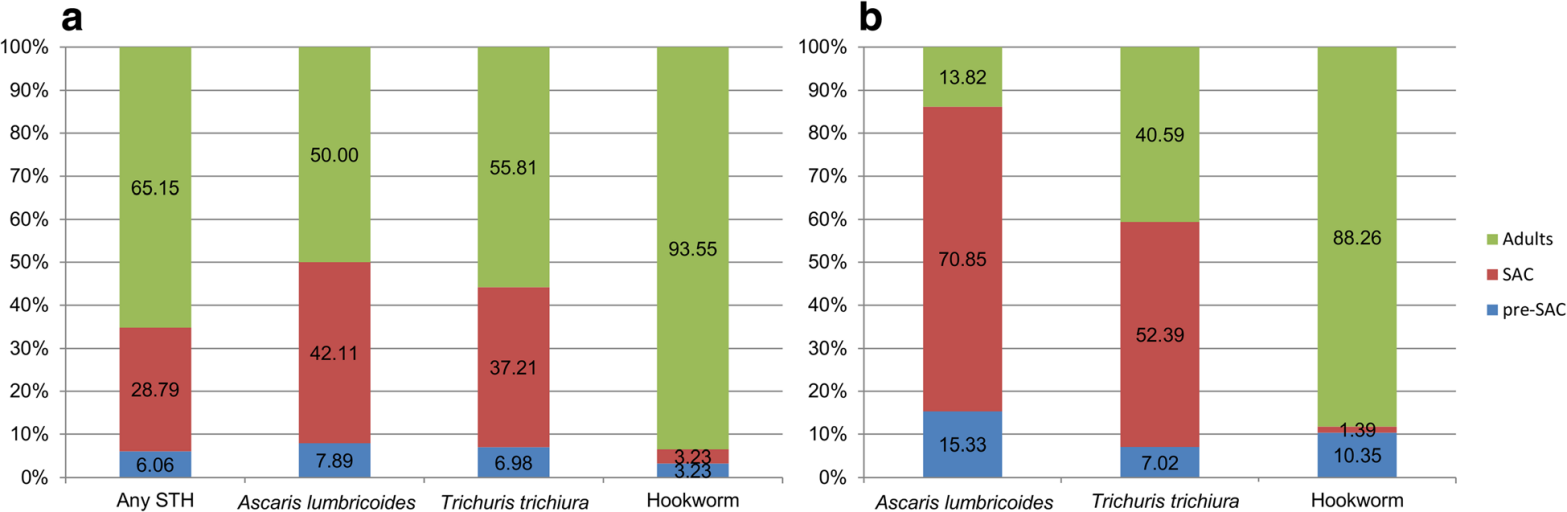
Burden of STH infection within age groups. **a** Proportion of positive individuals for each STH within age groups. **b** Proportion of total EPG of each STH within age groups. *Key*: Pre-SAC, preschool-aged children (2–4 years old); SAC, school-aged children (5–14 years old); Adults (15+ years old). Labels denote percentage within each STH

Prevalence of infection with at least one STH in adults (over 15 years old) was 25.75%. The most prevalent STH in adults was *T. trichiura* (14.37%), followed by hookworm (11.58%) and *A. lumbricoides* (5.66%). The difference in prevalence between adults and the other age groups was not statistically significant for overall STH prevalence (*χ* ^2^ = 3.24, *P* = 0.07), but was significant for each separate STH (*A. lumbricoides*: *χ* ^2^ = 6.99, *P* < 0.01; *T. trichiura*: *χ* ^2^ = 15.16, *P* < 0.0001; hookworm: *χ* ^2^ = 16.31, *P* < 0.0001). Within the adults, the only significant difference in prevalence between males and females was in hookworm (*A. lumbricoides*: *χ* ^2^ = 1.62, *P* = 0.20; *T. trichiura*: *χ* ^2^ = 0.43, *P* = 0.51; hookworm: *χ* ^2^ = 14.48, *P* < 0.001); males had a higher prevalence of hookworm (18.06%) compared to females (6.67%). Figure 3a shows the age breakdown of the STH-positive study participants alone. Of all the study participants infected with at least one STH, 65.15% were adults. Hookworm infections had the highest proportion of adults (93.55%) followed by *T. trichiura* infection (55.81%) and *A. lumbricoides* infection (50.00%). However, Fig. 3b shows the proportion of cumulative EPG by age group and STH species. Adults are the major contributors to the total hookworm (88.26%) EPG burden and contribute almost half of the total *T. trichiura* (40.59%) EPG burden. However, adults contribute very little to the total *A. lumbricoides* (13.82%) EPG burden.

## Discussion

There have been considerable advances in STH control in Myanmar over the last 15 years. The results of two WHO-assisted nationwide STH surveys published in 2004 [12] and 2013 [13] showed a marked decrease in STH prevalence in SAC. Furthermore, in 2015, national MDA coverage of SAC reported to the WHO PCT databank was higher than 95% [10]. However, there are currently no systematic monitoring and evaluation (M&E) programmes in place to assess changes in STH infection over time, to validate PCT coverage data or to examine individual compliance to treatment at each round of MDA. There have been no recent published assessments of STH infection in Myanmar that reported community-wide (pre-SAC, SAC and adults) quantitative infection intensity measurements, such as mean EPGs. The Ministry of Health of Myanmar is currently planning to put in place a more structured M&E programme to assess the impact of MDA on STH and LF.

The present study aimed to measure the burden of STH infection in communities that had been receiving MDA. The study villages chosen had received three years of MDA (starting in 2013) at the beginning of the study and four years of MDA by the end of the study. Results presented here, from the first survey in August 2015, revealed that 27.81% of participants remain infected with at least one STH. Similar STH prevalence were recently recorded in the Philippines [19], Cambodia [20] and Vietnam [21]. The prevalence of each STH in SAC was also higher than the last reported measurements in 2013 [13], indicating that more comprehensive M&E is required to accurately assess the national burden of STH. The data presented here provide valuable evidence that, at least in the delta area, STH prevalence has not substantially decreased since the last M&E activities in 2013 and that further work is required to elucidate the STH situation in the whole country. Subsequent reports will analyse the longitudinal data from all three surveys focussing on patterns of STH reinfection, the presence of and factors associated with predisposition to infection, and compliance to treatment by gender and age group.

A key finding of this study was that *T. trichiura* is the most prevalent STH. It has been well documented that albendazole is significantly less efficacious against *T. trichiura* than the other STH [22, 23]. A systematic review and meta-analysis by Keiser & Utzinger, 2008 [22] reported the cure rate (CR) of albendazole against *T. trichiura* to be just 43.6% (as opposed to 78.4% against hookworm and 93.9% against *A. lumbricoides*). Low drug efficacy against *T. trichiura* could have contributed to the limited decrease in prevalence over the period between the WHO nationwide surveys compared to *A. lumbricoides* and hookworm [12, 13]. Other possibilities such as benzimadazole drug resistance and inadequate drug absorption should also be explored in future M&E studies [24, 25].

The STH MDA programme in Myanmar is targeted at SAC, as recommended by the WHO [4], with the aim of reducing STH-related morbidity. However, there is an increasing focus on STH infection in adults as research and control goals turn towards the prospect of interrupting transmission [26–28]. The majority of STH infections in the study villages are harboured by adults (65.15%). Furthermore, most of the hookworm EPG burden within the community was harboured in adults. This is not surprising as it has been well documented that hookworm prevalence and intensity is consistently higher in adults [29]. It is surprising that 40.59% of the total *T. trichiura* EPG burden was in adults. This will in part be due to the fact that there are more adults in the study sample than children. Adults are clearly significant contributors to STH transmission, perhaps due to poor compliance to MDA. Studies in Laos [30, 31] and Malaysia [32] have also found similar STH prevalence in adults. Whilst treatment and monitoring of infection in children is of the utmost importance to control morbidity, adults should be included in M&E programmes. Adults are an important reservoir of infection and STH transmission control will not be achieved without greater focus on the older age groups.

It is important to note that individual compliance (actually ingesting the drug at each round of treatment) to MDA programmes is not well recorded or researched in any national MDA programme for which WHO records MDA coverage [33, 34]. Reported coverage may be high [10], but if this does not include monitoring of actual albendazole ingestion then the numbers may be inflated. If there is systematic non-compliance to MDA in the community, STH prevalence and intensity will not drop as rapidly as predicted when assuming full compliance [35] given that persistent non-compliers act as a reservoir of infection. We attempted to avoid non-compliance during the study by instructing the participants to ingest the albendazole immediately upon administration by the survey teams.

A limitation of this study is that only a single slide from a single stool sample per participant per survey was examined. Due to the low sensitivity of the Kato-Katz technique [36–38], there is a possibility that the prevalence of STH has been underestimated. Research on the use of quantitative polymerase chain reaction (qPCR) for testing stool samples has concluded that the qPCR technique is more sensitive than Kato-Katz [39, 40]. Faecal samples from the study described here have been stored for qPCR diagnostic testing. Further epidemiological research on STH should include a qPCR component for a more accurate assessment of prevalence and intensity. Another limitation is the possibility of selection bias from the inclusion criteria and from the characteristics of participants lost to follow-up. Due to ethical considerations, stool samples could not be taken from people who refused treatment. Therefore, the results may be biased towards participants who are more likely to be compliant to MDA and consequently have their STH infections treated more frequently, underestimating STH infection in the community. Unfortunately, loss to follow-up was high with only 67.45% of enrolled participants completing the study. The main reason given by participants for dropping out of the study was fatigue with the study methods, mainly citing the stool collection. For future STH studies, to ensure that sample size is maximised, it would be beneficial to make stool collection as easy as possible for the participants. This complication also provides an impetus for further developing new diagnostic techniques, such as ELISA [41, 42], that are reliant on blood samples instead of stool samples.

## Conclusions

Despite four years of SAC-targeted and community-wide MDA, STH transmission is continuing in the chosen study sites. STH infection was found in all age groups but the majority of infections were found in adults. Whilst the results presented here will be helpful to the national MDA programmes of Myanmar, further surveys are required in the different ecological zones to fully elucidate the nationwide epidemiological pattern of STH infection in Myanmar. Much higher prevalences might be expected in remote rural regions. There is an urgent need for the design and implementation of community wide M&E programmes.

## Additional files

Additional file 1: STROBE checklist. (TIFF 665 kb)

Additional file 2: Sample size back calculation. (DOC 85 kb)

Additional file 3: Figure S1. Study participation flow-chart. (TIFF 524 kb)

Additional file 4: Figure S2. Study methods flow-chart. (DOCX 17 kb)

Additional file 5: Figure S3. Age pyramids. (TIFF 465 kb)

## Abbreviations

CI: Confidence interval
CR: Cure rate
DALY: Disability-adjusted life year
DEC: Diethylcarbamazine citrate
EPG: Eggs per gram of faeces
GPS: Global positioning system
LF: Lymphatic filariasis
M&E: Monitoring and evaluation
MDA: Mass drug administration
MMK: Myanmar kyats
NTD: Neglected tropical diseases
PCT: Preventive chemotherapy
Pre-SAC: Preschool-aged children
qPCR: quantitative polymerase chain reaction
SAC: School-aged children
STH: Soil-transmitted helminths
STROBE: Strengthening the reporting of observational studies in epidemiology
WHO: World Health Organization.
