# Supplementary figures and images for: A cross-sectional survey of soil-transmitted helminthiases in two Myanmar villages receiving mass drug administration: epidemiology of infection with a focus on adults
Source: Parasit Vectors. 2017 Aug 4;10:374. doi: 10.1186/s13071-017-2306-2 (PMC5543579; doi:10.1186/s13071-017-2306-2)

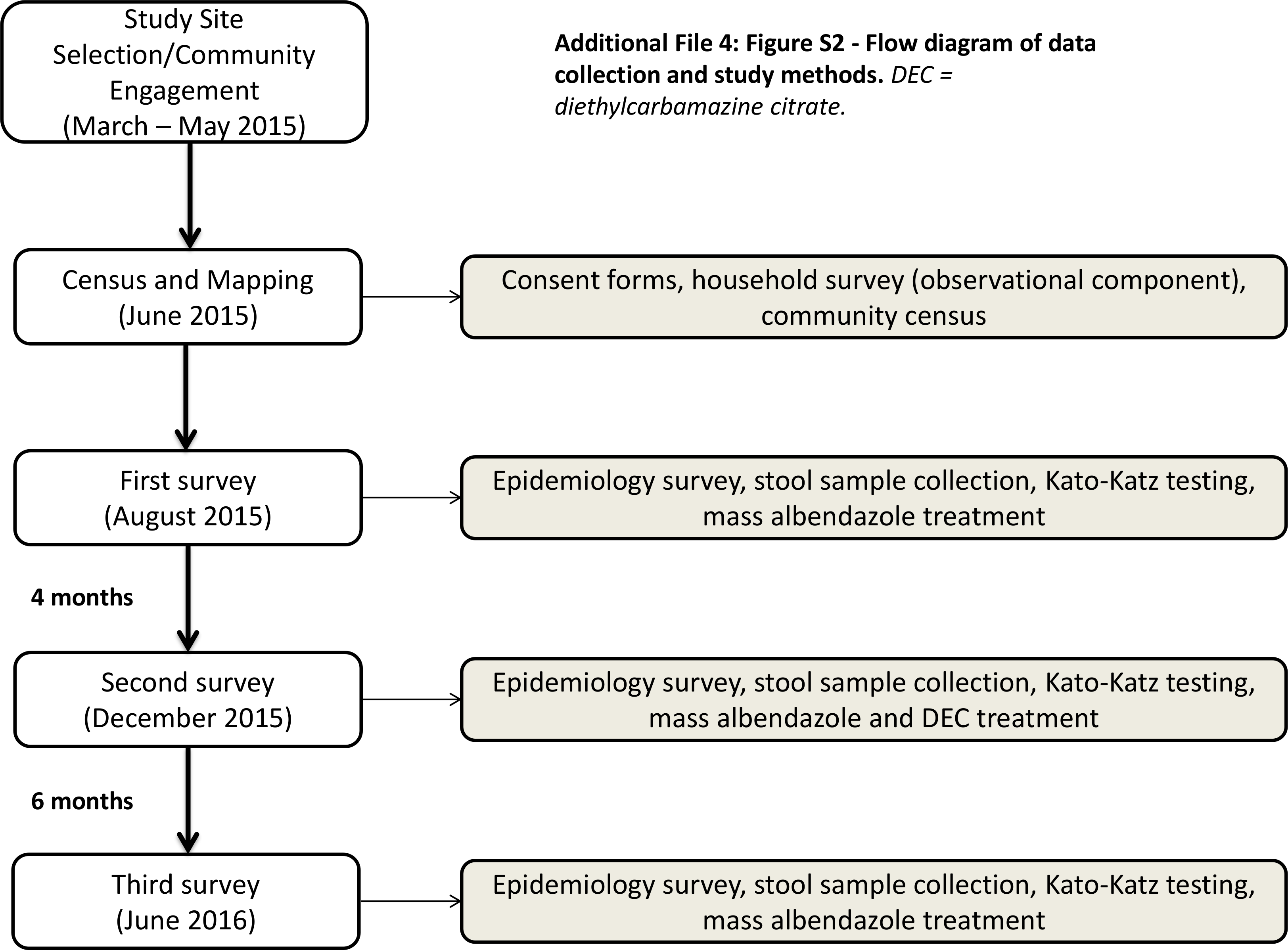

Supplement: Supplementary file 1 — STROBE checklist. (TIFF 665 kb) [file 13071_2017_2306_MOESM4_ESM.tif]

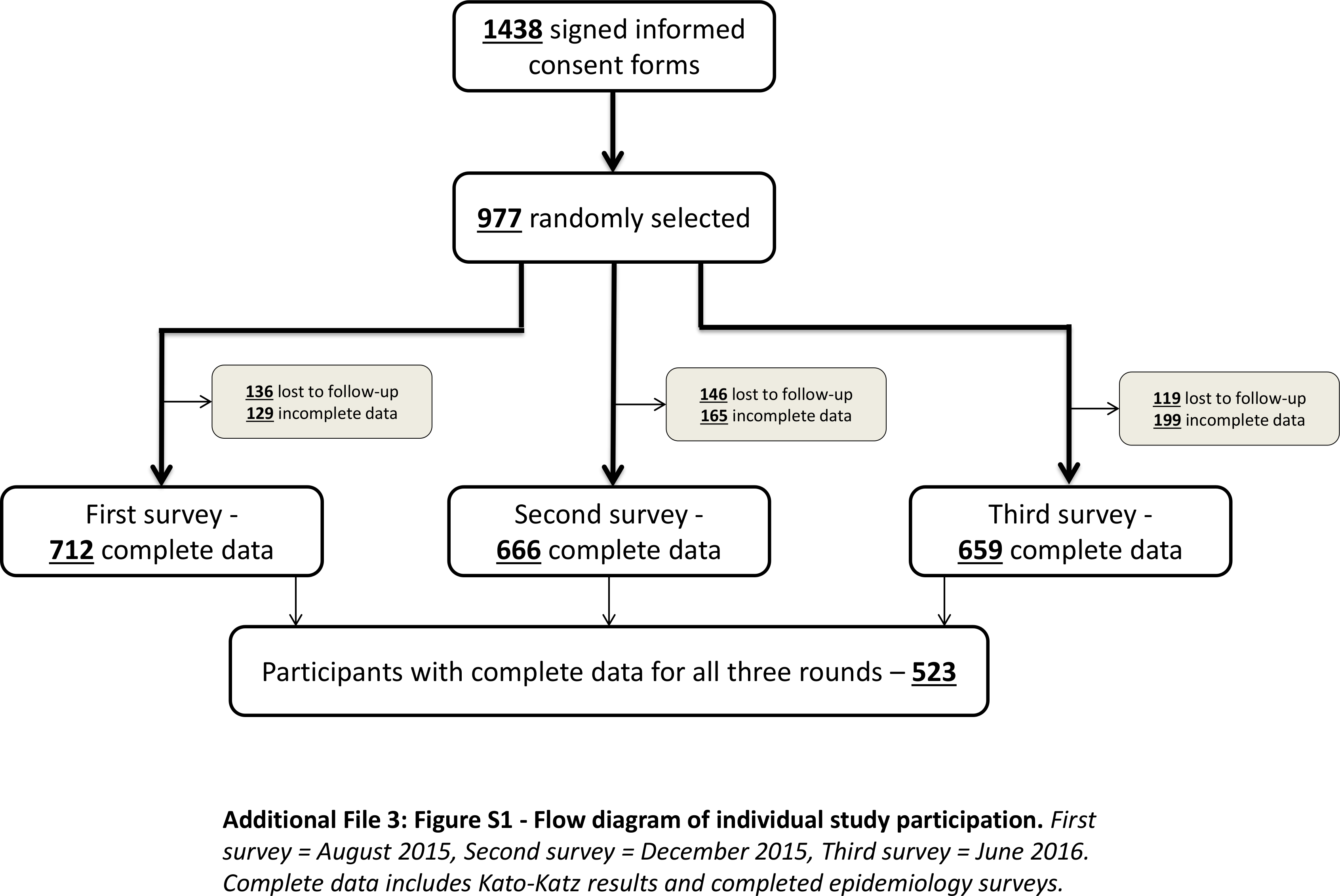

Supplement: Supplementary file 3 — Study participation flow-chart. (TIFF 524 kb) [file 13071_2017_2306_MOESM3_ESM.tif]

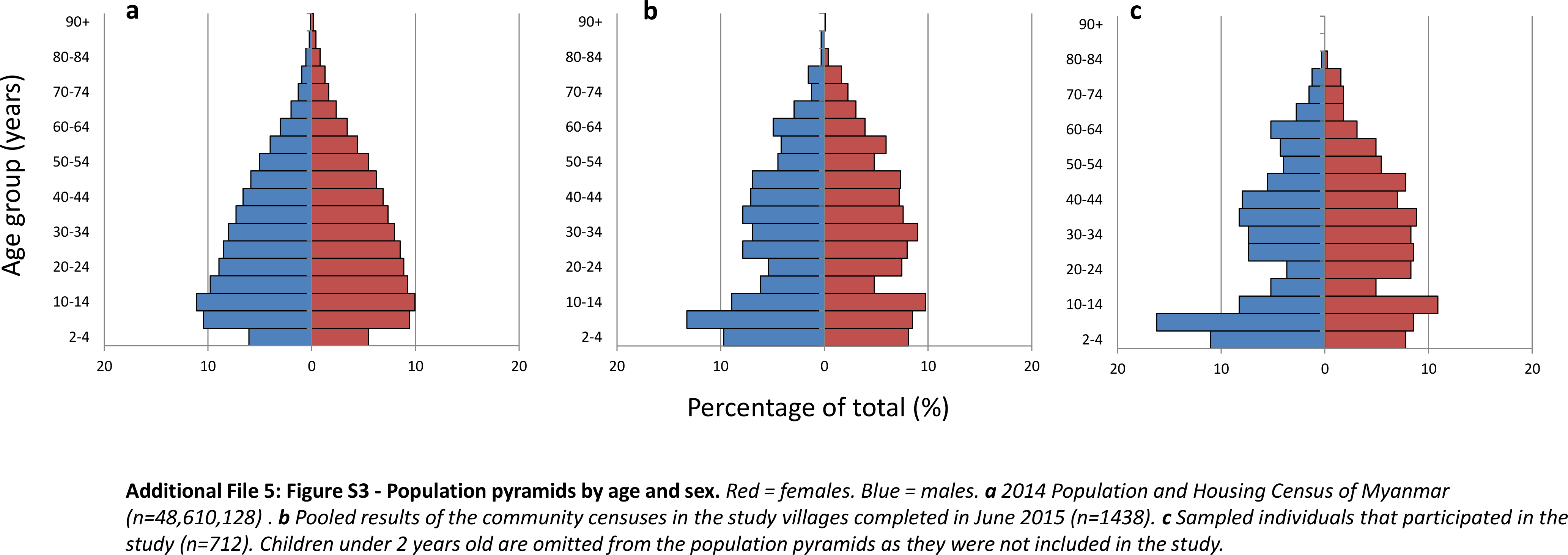

Supplement: Supplementary file 5 — Age pyramids. (TIFF 465 kb) [file 13071_2017_2306_MOESM5_ESM.tif]
