# Supplementary material for: A cross-sectional survey of soil-transmitted helminthiases in two Myanmar villages receiving mass drug administration: epidemiology of infection with a focus on adults
Source: Parasit Vectors. 2017 Aug 4;10:374. doi: 10.1186/s13071-017-2306-2 (PMC5543579; doi:10.1186/s13071-017-2306-2)
Supplement: Supplementary file 4 — Study methods flow-chart. (DOCX 17 kb) [file 13071_2017_2306_MOESM2_ESM.docx]

Additional File 2 – Sample size back calculation

At the beginning of the study, an ideal was sample size was calculated based purely on logistics; the maximum number of slides that the laboratory technicians could process over the allotted study period. After the study a sample size back calculation was formulated to determine the maximum margin of error derived from the final sample size and soil-transmitted helminth (STH) prevalence.

Equation 1 is the single proportion sample size calculation ^1^. $n$ = sample size, $z$ = percentage of the normal distribution corresponding to the required two-sided significance level (5% significance in this case), $m$ = margin of error, $p̂$ = proportion of interest (STH prevalence).

$${\left( 1 \right) n= \left( \frac{z}{m} \right)}^{2}*p̂ *(1-p̂)$$

This equation was rearranged to give the margin of error (Equation 2). The margin of error is the width of the 95% confidence interval around $p̂$.

$$\left( 2 \right) m= \frac{z}{\sqrt{n / (p̂*\left( 1-p̂ \right))}}$$

Equation 3 incorporates the study specific values (first survey).

$$\left( 3 \right) m= \frac{1.96}{\sqrt{712 / (0.2781*\left( 1-0.2781 \right))}}=0.0329$$

This gives $m$ = 0.0329 or 3.29%. This means that the 95% confidence interval around prevalence is 3.29% wide, or 1.65% above and below the prevalence. We deem that this is an acceptable margin of error.

1. Kirkwood BR, Sterne JAC, Kirkwood BR. *Essential Medical Statistics*. Blackwell Science; 2003.
